# Microglia/macrophages are ultrastructurally altered by their proximity to spinal cord injury in adult female mice

**DOI:** 10.1186/s12974-023-02953-0

**Published:** 2023-11-21

**Authors:** Marie-Kim St-Pierre, Fernando González Ibáñez, Antje Kroner, Marie-Ève Tremblay

**Affiliations:** 1 Axe Neurosciences, Centre de Recherche du CHU de Québec-Université Laval, Québec City, QC Canada; 2 Department of Molecular Medicine, Université Laval, Québec City, QC Canada; 3 Division of Medical Sciences, University of Victoria, 3800 Finnerty Road, Victoria, BC V8P 5C2 Canada; 4 Department of Neurosurgery, Medical College of Wisconsin, Milwaukee, WI USA; 5 Department of Microbiology and Immunology, Medical College of Wisconsin, Milwaukee, WI USA; 6 Clement J. Zablocki Veterans Affairs Medical Center, 5000 W. National Ave, Milwaukee, WI 53295 USA; 7 Department of Biochemistry and Molecular Biology, The University of British Columbia, Vancouver, BC Canada; 8 Department of Neurology and Neurosurgery, McGill University, Montréal, QC Canada; 9 Centre for Advanced Materials and Related Technology (CAMTEC) and Institute on Aging and Lifelong Health (IALH), University of Victoria, Victoria, BC Canada

**Keywords:** Spinal cord injury, Ultrastructure, Microglia, Peripheral macrophages, Phagocytosis, Cellular stress, Metabolism, Myelinated axons, Synaptic interactions, Mouse

## Abstract

Traumatic spinal cord injury can cause immediate physical damage to the spinal cord and result in severe neurological deficits. The primary, mechanical tissue damage triggers a variety of secondary damage mechanisms at the injury site which significantly contribute to a larger lesion size and increased functional damage. Inflammatory mechanisms which directly involve both microglia (MG) and monocyte-derived macrophages (MDM) play important roles in the post-injury processes, including inflammation and debris clearing. In the current study, we investigated changes in the structure and function of MG/MDM in the injured spinal cord of adult female mice, 7 days after a thoracic contusion SCI. With the use of chip mapping scanning electron microscopy, which allows to image large samples at the nanoscale, we performed an ultrastructural comparison of MG/MDM located near the lesion vs adjacent regions to provide novel insights into the mechanisms at play post-injury. We found that MG/MDM located near the lesion had more mitochondria overall, including mitochondria with and without morphological alterations, and had a higher proportion of altered mitochondria. MG/MDM near the lesion also showed an increased number of phagosomes, including phagosomes containing myelin and partiallydigested materials. MG/MDM near the injury interacted differently with the spinal cord parenchyma, as shown by their reduced number of direct contacts with synaptic elements, axon terminals and dendritic spines. In this study, we characterized the ultrastructural changes of MG/MDM in response to spinal cord tissue damage in mice, uncovering changes in phagocytic activity, mitochondrial ultrastructure, and inter-cellular interactions within the spinal cord parenchyma.

**Supplementary Information:**

The online version contains supplementary material available at 10.1186/s12974-023-02953-0.

## Introduction

Traumatic spinal cord injury (SCI) often leads to permanent neurological damage to motor, sensory and autonomous nervous systems in the affected individuals. Injuries are most frequently caused by accidents, resulting in contusive impact to the spinal cord and immediate tissue damage. This damage is further aggravated by secondary mechanisms, including excitotoxicity, edema, hemorrhage, and inflammation [1].

The immediate inflammatory response to injury is characterized by a local immune reaction, notably consisting of cytokine production and altered phenotypic states (e.g., involving morphology, function, molecular signature) of various central nervous system (CNS) resident cells, followed by an invasion of peripheral innate immune cells [2, 3]. In the last years, the role of both peripheral, monocyte-derived macrophages (MDM) and CNS resident macrophages (microglia; MG) after SCI has gained particular attention. Microglia are ontogenetically different from MDMs, arising from the yolk sac around embryonic days 8–9.5 after which they populate the CNS in mice [4]. They perform surveillance of the CNS parenchyma under normal physiological conditions and quickly respond to injuries for instance by migrating towards the site of damage and redirecting their processes to seal the lesion [5, 6]. The purinergic P2Y12 receptor-mediated MG response is essential for containing and controlling CNS lesions [7]. Microglial depletion with colony-stimulating factor 1 receptor (CSF1R) inhibitors administered before or early after a contusion SCI in adult mice resulted in large, disorganized lesions and impaired functional recovery, supporting the important role of MG in mediating scar interface formation and lesion containment after SCI [8, 9]. By contrast, other groups have demonstrated improved recovery and tissue preservation after prolonged CSF1R treatment in young adult mice with a thoracic vertebra (T)9 SCI [10, 11]. Overall, the early, local immune response is considered to be essential for the removal of cellular debris and containment of the lesion [8, 9, 12], but a prolonged and ultimately damaging inflammatory response is observed after SCI. Unlike in other tissues, inflammation within the spinal cord does not resolve, both in rodent models and post-mortem tissue samples from patients with SCI, where changes in MG or MDMs remain present for extended time frames [13–15]. An important role performed by MG/MDM is the phagocytosis of tissue components after injury. Greenhalgh and David showed that MG contact the damaged axons early after a moderate T11 contusion injury in lys-EGFP-knock-in mice in which MDM but not MG express EGFP [16]. Until MDM invade the injured spinal cord 3 days after SCI, MG were the main cell type containing auto-fluorescent lipofuscin, indicative of undigested or residual phagocytosed materials. Histologically, lipofuscin was not detectable anymore in MG at 7, 14, 28 and 42 day post-injury when phagocytosis was taken over by MDM [16]. Due to their ability to perform phagocytosis during their lifespan which is longer than that of MDM, MG demonstrate an increased ability to digest and break down tissue debris compared to MDM [16, 17].

To provide insights into the roles of MG/MDM after SCI, we performed an ultrastructural analysis of their structural and functional characteristics (e.g., phagocytosis, metabolism) in the injured spinal cord tissue 7 days after a T11 contusion injury in adult female mice. While electron microscopy investigations post-SCI previously revealed at the nanoscale changes in axon and myelin morphology [18], blood–brain barrier alterations [19], vascular structures [20], or cavity formation and overall cell composition at chronic time points [21] in rodent and primate models, to our knowledge, this study is the first to characterize the ultrastructural alterations of MG/MDM. We focused our analysis on features providing novel insights into the roles of MG/MDM after SCI, in particular their interaction with the parenchyma, the number and composition of phagosomes and, finally, their mitochondrial number and integrity. The role of mitochondria upon SCI is multifold [22]: In the early stages after injury, glutamate release from MG [23] and damaged neurons results in their excessive intracellular calcium concentration and subsequent mitochondrial dysfunction and cell death [24]. In addition, mitochondria are major producers of reactive oxygen species (ROS), which contribute to the regulation of cell death mechanisms and influence the inflammatory response [22]. By comparing the characteristics of MG/MDM between the lesion site and uninjured tissue in mice 7 days after a T11 contusion injury, we detected abnormal interactions of MG/MDM with degraded axons close to the lesion site and a high proportion of MG/MDM displaying phagosomes, containing cell debris and myelinated elements, and finally a high number of altered mitochondria, an ultrastructural sign of cellular stress, near the injury.

## Methods

### Ethics approval and consent to participate

All animal experiments were approved and performed according to the guidelines of the Institutional Care and Use Committees of the Clement J. Zablocki VA Medical Center and the Medical College of Wisconsin.

### Mice

Eight-week-old C57BL/6 female mice were obtained from Charles River Laboratories and housed under conventional conditions on a 12-h light–dark cycle with 20–22 °C room temperature, humidity between 30% and 70% and ad libitum access to food and water. Environmental enrichment was provided in form of Enviro-dri^®^ and huts in the cages. Mice were housed in groups of three, cages were cleaned twice weekly, while mice were handled during cage changes and behavioral assessments. All animals received routine animal care including regular chow and acidified water.

### Spinal cord contusion injury

For the induction of SCI, mice were deeply anesthetized with isoflurane (4% induction, 2.5% maintenance). After routine skin preparation, sterile technique was used for the procedure. After a T11 laminectomy, a moderate contusion (40 kdyne) was induced with the Infinite Horizon Impactor device (Precision Systems and Instrumentation, LLC, Lexington, KY, USA). The average tissue displacement was 317 ± 58 μm. Wounds were closed with absorbable Vicryl sutures and skin clips that were removed 7 day post-op. Analgesia was achieved by subcutaneous carprofen injections (5 mg/kg) twice daily on the surgery day and for 3 day post-injury, with bladders manually expressed twice daily until normal micturition resumed. Seven day post-injury, mice were euthanized with an overdose of Phenytoin/Pentobarbital (120 mg/kg), and transcardially perfused with phosphate-buffered saline (PBS; 50 mM, pH 7.4), followed by perfusion fixation with cold 4% paraformaldehyde (PFA) and 0.25% glutaraldehyde. One-mm long segments of spinal cord tissue, centering on the SCI injury site, were dissected. Fifty µm-thick coronal sections of the spinal cords were then obtained using a vibratome (Leica VT1000s) in ice-cold PBS and kept at – 20 °C until further experimentation in cryoprotectant [20% (v/v) glycerol, 20% (v/v) ethylene glycol in PBS].

### Tissue post-fixation for scanning electron microscopy

Spinal cord sections from the lower thoracic region containing the region of interest (dorsal column) obtained from three mice were selected for scanning electron microscopy (SEM) experiments. Only sections located near the spinal cord injury (less than 1 mm away) were processed for imaging. Sections were washed with phosphate buffer (PB, 100 Mm, pH 7.4) then incubated for 1 h in a solution comprising equal volumes of 4% osmium tetroxide (EMS, Pennsylvania, USA, cat# 19190) and 3% potassium ferrocyanide (Sigma-Aldrich, Ontario, Canada, cat# P9387) diluted in PB. Following washes in double-distilled water, the sections were incubated in a filtered 1% thiocarbohydrazide solution (diluted in double-distilled water; Sigma-Aldrich, Ontario, Canada, cat# 223220) for 20 min, then in 2% aqueous osmium tetroxide for 30 min. The sections were dehydrated in increasing concentrations of ethanol for 10 min each (2 × 35%, 1 × 50%, 1 × 70%, 1 × 80%, 1 × 90%, 3 × 100%) and washed in propylene oxide (Sigma-Aldrich, #cat 110205-18L-C) 3 times 10 min. The spinal cord tissues were embedded overnight in Durcupan resin (20 g component A, 20 g component B, 0.6 g component C, 0.4 g component D; Sigma Canada, Toronto, cat# 44610) and delicately placed for flat-embedding on fluoropolymer films (ACLAR^®^, Pennsylvania, USA, Electron Microscopy Sciences, cat# 50425-25) the next day. The montage was kept in a convection oven at 55 °C for 5 days to allow for resin polymerization.

Following resin polymerization, the region of interest contained in resin (dorsal column) was excised from the embedded sections and glued onto resin blocks for ultramicrotomy using a Leica ARTOS 3D. Sections from two to four levels per animal (6–8 µm apart) were cut at a thickness of 73 nm and placed onto silicon wafers for SEM imaging using a Zeiss Crossbeam 350 SEM operating at a 1.4 kV voltage and 1.2 nA current. Images of the ultrathin sections were first acquired at a resolution of 25 nm per pixel to allow for the identification of MG/MDM cell bodies with relation to the SCI site (Additional file 1: Fig. S1) [25–27]. This allowed the creation of massive mosaics of the sections with ultrastructural resolution. This enabled the identification and differentiation of the injury zone vs the healthy surrounding tissue (Additional file 1: Fig. S1E–G). The lesion site was ultrastructurally identified by the clear degradation of parenchymal elements, such as high presence of degraded and redundant myelin and apoptotic cells, abundance of cellular stress markers in parenchymal elements, such as dystrophic mitochondria and autophagosomal vesicles, and accumulation of lipid droplets and debris membranes in the intercellular space. MG/MDM located near the lesion area (directly contacting degraded myelinated axons or surrounded by parenchyma with clear signs of dystrophy or myelin alterations) and far (in proximity to the white matter of the dorsal column adjacent to the lesion surrounded by parenchyma without any signs of dystrophy or myelin alterations but observed in the same ultrathin section) were next imaged at a resolution of 5 nm per pixel for ultrastructural analyses. Images were stitched and exported as tifs using the software Zeiss Atlas 5 (Fibics, Ontario, Canada).

### Ultrastructural analysis of MG/MDM located far vs near the SCI site

Images of 10–14 MG/MDM from each animal (*n* = 3 animals; 37–42 MG/MDM per location, far from vs near the injury site) at a resolution of 5 nm per pixel were analyzed. All images were blinded to the experimental conditions. We analyzed a total sample size of 79 MG/MDM cell bodies which was determined to be sufficient to obtain statistical power based on the software G*Power V3.1 (effect size of 0.9; power of 0.9 estimated at 60 MG/MDM) [25, 27, 28]. We did not perform immunostaining to distinguish MG/MDM as we wanted to further investigate the presence of glycogen granules within their cytoplasm [27] and the possible presence of dark MG [27, 29]. Dark MG have been described in different pathological contexts in mice including models of demyelination [30, 31]. We wondered if this would be the case in this model. MG/MDM were instead identified based on their distinct ultrastructural features, including their hetero- and euchromatin pattern, the presence of long and narrow stretches of endoplasmic reticulum (ER) and unique distribution of organelles throughout their cytoplasm [26, 32]. Dark MG are differentiated from MG by an electron-dense cytoplasm, loss of nuclear heterochromatin pattern and prevalent presence of ultrastructural markers of cellular stress [27]. The quantitative analysis of MG’s intracellular content and their direct interactions with parenchymal elements was previously described in [26, 27, 32–35].

For parenchymal investigation, MG/MDM interactions with myelinated axons, both non- and degraded, were assessed. Non-degraded myelinated axons were identified by their electron-dense sheaths surrounding the axonal cytoplasm, while myelinated axons were classified as degraded if the myelin sheaths were degraded and/or if the myelinated axons were swollen or showed signs of dystrophy (e.g., presenting a dark cytoplasm) [36, 37]. The ratio of contacts with degraded myelinated axons over all myelinated axons was calculated. The number of MG/MDM making a direct contact with a degraded myelinated axons was also determined. Axon terminals were characterized by their numerous circular synaptic vesicles (about 40 nm in diameter) while dendritic spines were only positively identified if they were located next to an axon terminal and possessed a post-synaptic density (electron-dense area) [26, 35, 38].

For MG/MDM intracellular content analysis, we assessed the presence of ultrastructural markers of phagolysosomal activity (partially digested phagosomes, myelin containing phagosomes, fully digested phagosomes, autophagosomes, primary lysosomes, secondary lysosomes, tertiary lysosomes), cellular stress (altered mitochondria, dilated ER) and alteration to other organelles (lipid bodies, elongated mitochondria, non-altered mitochondria, glycogen granules). Immature (primary and secondary) lysosomes were identified by their homogeneous (primary) or heterogeneous (secondary) appearance with the presence of electron-dense granules within. Tertiary lysosomes were differentiated from secondary lysosomes by the additional presence of residual lipid bodies and fully or partially digested phagosomes [26, 37, 39, 40]. Partially and fully digested phagosomes were categorized based on their defined circular outline with an electron-lucent interior containing (partially digested) or not (fully digested) cellular elements [26, 40]. We further examined phagosomes specifically presenting features of myelinated elements due to the location of the MG/MDM cell bodies within the white matter. Autophagosomes were recognized by their circular double-membrane containing elements with an interior that had the same appearance (electron-density) as the cytoplasm it came from [26, 27].

Endoplasmic reticulum were identified by their long and narrow stretches located throughout the cytoplasm, while their cisternae were determined to be dilated if their width was beyond 100 nm [26, 32, 34, 41, 42]. The ratio of dilated ER cisternae over non-dilated counterparts was calculated. Mitochondria were distinguished by their electron-dense double membrane and interior containing several cristae structures [26, 32, 39, 43, 44]. Mitochondria were defined as altered if they possessed one of the following; degraded inner or outer membrane as identified by electron-lucent patches, swollen appearance, enlarged and electron-lucent cristae, or “holy shape” shown by mitochondria enwrapping themselves [26, 29, 41, 45]. Elongated mitochondria were positively identified if their length measured more than 1000 nm [26, 33]. The proportion of cells containing at least one element (relative percentage) was calculated for altered mitochondria and elongated mitochondria. The ratio of altered mitochondria over all mitochondria was also calculated. We additionally investigated the presence of glycogen granules, a carbohydrate storage that is defined ultrastructurally as 20–42 nm diameter electron-dense puncta [46]. These were recently shown using SEM to be present within the cytoplasm of MG located near dystrophic neurites and amyloid beta plaques in a mouse model of Alzheimer’s disease pathology [27].

We further analyzed using the software ImageJ the shape of MG/MDM cell bodies by tracing the cytoplasmic and nuclear membranes with the “Freehand tool”, examining area, perimeter, solidity, aspect ratio and circularity. Aspect ratio was calculated by dividing the height over the width of the cell body while circularity was determined by multiplying 4π times the area over the perimeter squared [34, 37, 47, 48]. Both measurements provide information on the elongation state of the cell (e.g., the closer the value is to 0, the more elongated is the cell body based on circularity) [37, 47, 48].

### Statistical analysis

Statistical tests were performed with the Prism 9 software (v.9.2.0 GraphPad). Quantitative ultrastructural data were tested for their normality using the Shapiro–Wilk test. Only the circularity data passed normality and was compared between locations (far from vs near SCI site) using an unpaired two-tailed Student’s *t* test with a Welsh’s correction. All other data did not pass normality and they were compared between locations using a non-parametric Mann–Whitney test. Data are expressed as mean ± standard error of mean (SEM). The sample size (*n*) refers to individual MG/MDM as performed in previous ultrastructural MG studies to take into account heterogeneity within the MG populations [27, 28, 34, 42, 44, 49–51]. Statistically significant differences are reported as *p < 0.05, **p < 0.01, ***p < 0.001 and ****p < 0.0001.

## Results

### MG/MDM near the injury site present altered interactions with the parenchymal elements

**Fig. 1:**
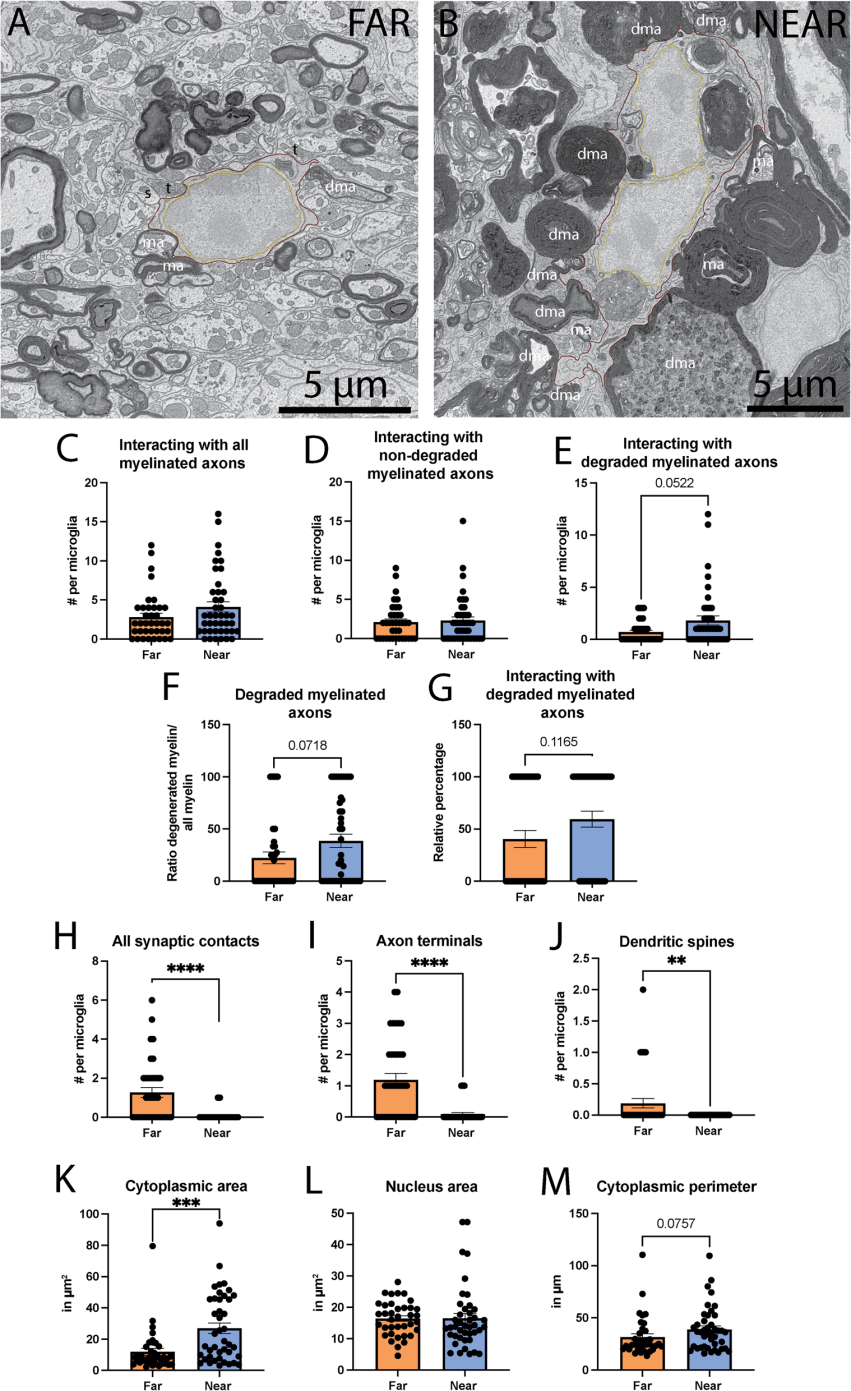
Parenchymal interactions of MG/MDM far from and near the SCI site in the dorsal column. Representative 5 nm per pixel scanning electron microscopy (SEM) images of MG/MDM far (**A**) and near (**B**) the spinal cord injury (SCI) site in the dorsal column of lower thoracic spinal cord of 8-week-old female SCI mice. Quantitative graphs representing the number of direct contacts with myelinated axons (**C**), non-degraded myelinated axons (**D**) and degraded myelinated axons (**E**), the ratio of direct contacts with degraded myelinated axons over all myelinated axons (**F**), the relative percentage of cells positive for at least one contact with a degraded myelinated axon (**G**), the number of direct contacts with all synaptic contacts (**H**), axon terminals (**I**) and dendritic spines (**J**). The area of the cytoplasm (**K**), the nucleus (**L**) and the cytoplasmic perimeter (M) are shown. Data are expressed as individual dots and are shown as means ± SEM. *p < 0.05, using a non-parametric Mann–Whitney test. Statistical tests were performed on *n* = 10–14 MG/MDM per animal with *N* = 3 mice/group, for a total of 79 cell bodies analyzed. Red outline = cytoplasmic membrane, yellow outline = nuclear membrane, ma = non-degraded myelinated axon, dma = degraded myelinated axon, t = axon terminal, s = dendritic spine

We first investigated the parenchymal interaction of MG/MDM far from vs near the injury site in the dorsal column of the lower thoracic region of a SCI female mouse model. MG/MDM near the injury site did not significantly interact more with non-degraded myelinated axons compared to MG/MDM far from the injury site (Far 2.108 ± 0.3773 contact per MG/MDM vs Near 2.310 ± 0.4641 contact per MG/MDM, *p* = 0.9661), but there was a tendency for MG/MDM near vs far from the injury site to increase their interaction with degraded myelinated axons (Far 0.7027 ± 0.1683 contact per MG/MDM vs near 1.810 ± 0.4287 contact per MG/MDM, *p* = 0.0522) (Fig. 1C–E). We further investigated the ratio of contacts with degraded myelinated axons over all contacts with myelinated axons in MG/MDM far from vs near the injury site and found a tendency for an increase in the ratio of degraded myelinated axons in the MG/MDM near the injury site (Far 22.34 ± 5.694% of degraded myelinated axons vs Near 38.58 ± 6.355% of degraded myelinated axons, *p* = 0.0718). In addition, a tendency for more MG/MDM displaying at least one contact with a degraded myelinated axon was observed near vs far from the injury site (Far 40.54 ± 8.183% of MG/MDM vs Near 59.12 ± 7.666% of MG/MDM, *p* = 0.1165) (Fig. 1F, G). These data indicate that MG/MDM located near the injury site are more often in contact with degraded myelinated axons compared to non-degraded myelinated axons.

MG dynamically interact with synaptic elements throughout the lifespan, in health and in disease, where they can alter their activity and plasticity via various mechanisms [i.e., trogocytosis or “nibbling” [52] and phagocytosis of axon terminals and dendritic spines, as well as physical separation of both synaptic elements [53–56]]. Therefore, we analyzed the direct interaction of MG/MDM with axon terminals and dendritic spines far from vs near the injury site. When we first examined all synaptic contacts (axon terminals and dendritic spines combined), MG/MDM far from the injury site contacted significantly more of these synaptic elements compared to MG/MDM near the injury site (Far 1.270 ± 0.2589 synaptic contact per MG/MDM vs Near 0.07143 ± 0.04022 synaptic contact per MG/MDM, *p* < 0.0001). Individually analyzing contacts with axon terminals or dendritic spines revealed an increased interaction with MG/MDM located far from compared to near the injury site (Far 1.189 ± 0.2081 contact with axon terminals per MG/MDM vs Near 0.09524 ± 0.04584 contact with axon terminals per MG/MDM, *p* < 0.0001; Far 0.1892 ± 0.07591 contact with dendritic spines per MG/MDM vs Near 0.000 ± 0.000 contact with dendritic spines per MG/MDM, *p* = 0.0084) (Fig. 1H–J). These differences were not due to a reduction in the area or perimeter of the cells. In fact, we observed a significantly increased cytoplasmic area (Far 11.94 ± 2.210 µm^2^ vs Near 26.95 ± 3.352 µm^2^, *p* = 0.0005), as well as a tendency toward an increased perimeter (Far 31.71 ± 3.048 µm vs Near 38.96 ± 3.206 µm, *p* = 0.0757) for MG/MDM located near vs far from the injury site (Fig. 1K–M). Combined, these results highlight the reduced interaction of MG/MDM with axon terminals and dendritic spines near the injury site.

### MG/MDM near the injury site comprise more phagosomes, specifically containing myelinated axons, within their cytoplasm

**Fig. 2:**
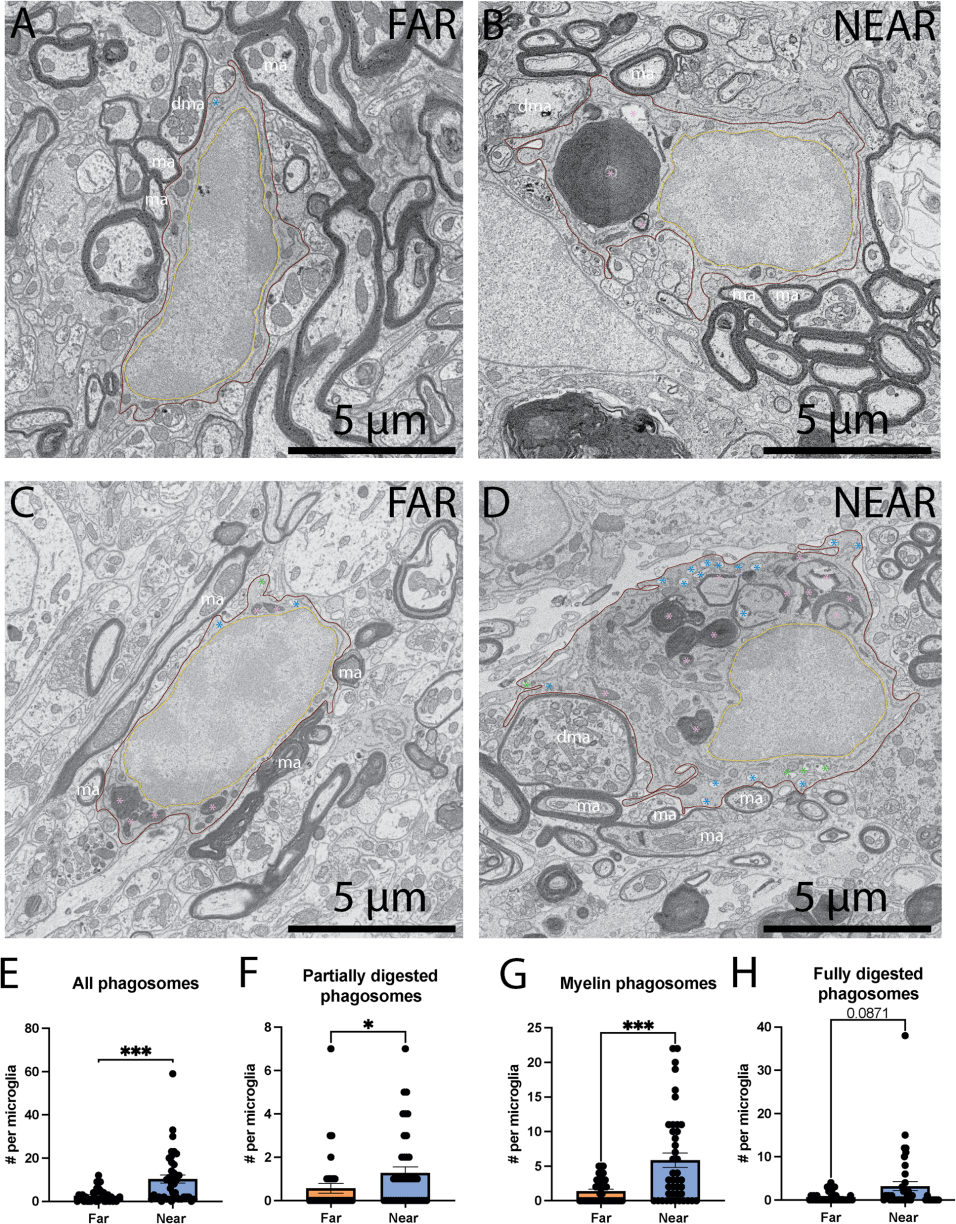
Phagocytic characteristics of MG/MDM far from and near the spinal cord injury (SCI) site in the dorsal column. Representative 5 nm per pixel scanning electron microscopy (SEM) images of MG/MDM far (**A**, **C**) and near (**B**, **D**) the SCI site in the dorsal column of lower thoracic spinal cord of 8-week-old female mice that received a contusion injury. Quantitative graphs representing the number of phagosomes within the MG/MDM cytoplasm (**E**), the number of partially digested phagosomes (**F**), phagosomes that are myelinated axons (**G**) and fully digested phagosomes (**H**). Data are expressed as individual dots and are shown as means ± SEM. *p < 0.05, using a non-parametric Mann–Whitney test. Statistical tests were performed on *n* = 10–14 MG/MDM per animal with *N* = 3 mice/group, for a total of 79 cell bodies analyzed. Red outline = cytoplasmic membrane, yellow outline = nuclear membrane, ma = non-degraded myelinated axon, dma = degraded myelinated axon, blue asterisk = fully digested phagosome, pink asterisk = myelin phagosome, green asterisk = partially digested phagosome

After SCI, MG/MDM have been shown to phagocytose parenchymal materials, notably degenerated myelin [57–60]. We therefore analyzed the intracellular content of MG/MDM, investigating particularly their phagolysosomal pathway. While we did not observe any differences in the number of mature and immature lysosomes in MG/MDM far from vs near the injury site (Table 1), we observed a significant increase in the number of phagosomes near vs far from the injury site (Far 2.649 ± 0.5100 phagosomes per MG/MDM vs Near 10.33 ± 1.829 phagosomes per MG/MDM, *p* = 0.0002) (Fig. 2E). We then further categorized phagosomes based on their content (myelinated elements), and whether they were partially or fully digested phagosomes, to determine which category of phagosomes was driving this significant difference. MG/MDM near the injury site contained significantly more myelinated elements within their cytoplasm (Far 1.400 ± 0.2722 myelinated elements per MG/MDM vs near 5.857 ± 1.024 myelinated elements per MG/MDM, *p* = 0.0008) (Fig. 2G), alongside significantly more partially digested phagosomes (Far 0.5676 ± 0.2210 partially digested phagosomes per MG/MDM near 1.286 ± 0.2668 partially digested phagosomes per MG/MDM, *p* = 0.0107) compared to far from the injury site (Fig. 2F). We also observe a tendency for more fully digested phagosomes in MG/MDM near compared to far from the injury site (Far 0.6757 ± 0.1820 fully digested phagosomes per MG/MDM vs near 3.190 ± 1.050 fully digested phagosomes per MG/MDM, *p* = 0.0871) (Fig. 2H). While myelinated axons are highly affected by contusion injury in the dorsal column, shown by the increase interaction of MG/MDM with degraded myelinated axons, our data further suggest that MG/MDM phagocytose these elements.

**Table 1:** Absolute ultrastructural analysis of MG/MDM far from and near the SCI injury site in the lower thoracic dorsal column of 8-week-old SCI female mice

	Far SCI Mean ± SEM (Min–Max)	Near SCI Mean ± SEM (Min–Max)
Primary lysosomes (*n*)	0.000 ± 0.000 (0.000–0.000)	0.02381 ± 0.02381 (0.000–1.000)
Secondary lysosomes (*n*)	0.02500 ± 0.02500 (0.000–1.000)	0.07143 ± 0.04022 (0.000–1.000)
Tertiary lysosomes (*n*)	0.05405 ± 0.05405 (0.000–2.000)	0.07143 ± 0.05272 (0.000–2.000)
All lysosomes (*n*)	0.08108 ± 0.05976 (0.000–2.000)	0.1667 ± 0.06745 (0.000–2.000)
Lipid bodies (*n*)	0.05405 ± 0.03769 (0.000–1.000)	0.1190 ± 0.05058 (0.000–1.000)
Non-altered mitochondria (*n*)*	7.459 ± 1.458 (0.000–46.00)	11.76 ± 1.734 (0.000–60.00)
Altered mitochondria (*n*)*	0.7750 ± 0.2594 (0.000–9.000)	1.326 ± 0.2711 (0.000–9.000)
Altered mitochondria (%)	6.768 ± 1.805 (0.000–37.50)	13.76 ± 3.508 (0.000–100.0)
Elongated mitochondria (*n*)	0.4250 ± 0.1285 (0.000–4.000)	0.4783 ± 0.1193 (0.000–4.000)
Elongated mitochondria (%)	6.859 ± 3.747 (0.000–100.0)	1.970 ± 0.5291 (0.000–12.50)
All mitochondria (*n*)*	8.622 ± 1.777 (0.000–59.00)	13.50 ± 1.949 (0.000–65.00)
Non-dilated endoplasmic reticulum (*n*)***	13.41 ± 1.887 (2.000–65.00)	28.79 ± 2.8383 (2.000–82.00)
Dilated endoplasmic reticulum (*n*)	0.2432 ± 0.08133 (0.000–2.000)	0.8810 ± 0.2752 (0.000–9.000)
All endoplasmic reticulum (*n*)***	13.65 ± 1.890 (2.000–65.00)	29.67 ± 3.531 (2.000–85.00)
% Dilated endoplasmic reticulum (*n*)	2.187 ± 0.7448 (0.000–16.67)	2.645 ± 0.7405 (0.000–22.22)
Partially digested phagosomes (*n*)*	0.5676 ± 0.2210 (0.000–7.000)	1.286 ± 0.2668 (0.000–7.000)
Fully digested phagosomes (*n*)	0.6757 ± 0.1820 (0.000–4.000)	3.190 ± 1.050 (0.000–38.00)
Myelin phagosomes (*n*)***	1.400 ± 0.2722 (0.000–5.000)	5.857 ± 1.024 (0.000–22.00)
All phagosomes (*n*)***	2.649 ± 0.5100 (0.000–12.00)	10.33 ± 1.829 (0.000–59.00)
Association with non-degraded myelinated axons (*n*)	2.108 ± 0.3773 (0.000–9.000)	2.310 ± 0.4641 (0.000–15.00)
Association with degraded myelinated axons (*n*)	0.7027 ± 0.1683 (0.000–3.000)	1.810 ± 0.4287 (0.000–12.00)
Association with all myelinated axons (*n*)	2.811 ± 0.4945 (0.000–12.00)	4.119 ± 0.6468 (0.000–16.00)
Association with degraded myelin (%)	22.34 ± 5.694 (0.000–100.0)	38.58 ± 6.355 (0.000–100.0)
Axon terminals (*n*)****	1.189 ± 0.2081 (0.000–4.000)	0.09524 ± 0.04584 (0.000–1.000)
Dendritic spines (*n*) **	0.1892 ± 0.07591 (0.000–2.000)	0.000 ± 0.000 (0.000–0.000)
All synaptic contacts (*n*)****	1.270 ± 0.2589 (0.000–6.000)	0.07143 ± 0.04022 (0.000–1.000)
Glycogen granules (%)	0.000 ± 0.000 (0.000–0.000)	9.524 ± 4.584 (0.000–100.0)
Autophagosomes (*n*)	0.1081 ± 0.06465 (0.000–2.000)	0.3571 ± 0.1732 (0.000–7.000)
Cell area (µm^2^)*	28.36 ± 2.737 (10.56–107.6)	43.44 ± 4.287 (12.77–123.2)
Cytoplasmic area (µm^2^)***	11.94 ± 2.210 (2.242–79.54)	26.95 ± 3.352 (3.143–94.07)
Nucleus area (µm^2^)	16.42 ± 0.9003 (4.500–28.08)	16.49 ± 1.568 (5.122–47.20)
Cell perimeter (µm)	31.71 ± 3.048 (13.75–110.4)	38.96 ± 3.206 (15.97–109.4)
Circularity (a.u.)	0.4457 ± 0.03066 (0.08600–0.7900)	0.4129 ± 0.02687 (0.1280–0.7740)
Aspect ratio (a.u.)	2.217 ± 0.1408 (1.034–4.942)	2.003 ± 0.1251 (1.085–4.948)
Solidity (a.u.)	0.7935 ± 0.02276 (0.3790–0.9680)	0.7852 ± 0.02001 (0.4860–0.9540)

*n* number, *a.u.* arbitrary unit

The *p* values of statistically significant tests are highlighted with an asterisk symbol. Data reported is expressed as means ± SEM as well as the minimum and maximum values obtained. *p < 0.05, **p < 0.01, ***p < 0.001, ****p < 0.0001 using a non-parametric Mann–Whitney test for data that did not pass normality (using Shapiro–Wilk test) and parametric *t* test with Welsh’s correction. Statistical tests were performed on *n* = 10–14 MG/MDM per animal with *N* = 3 mice/group, for a total of 79 cell bodies analyzed

### MG/MDM near the SCI injury site contain more mitochondria with an altered ultrastructure

Phagocytosis is an energy demanding process [61]. Previous studies have demonstrated an increase in ROS following SCI injury [62], alongside altered mitochondrial function and ER stress [63–65]. Therefore, we investigated in MG/MDM ultrastructural features associated with cellular stress, including the presence of dark cytoplasm and nucleoplasm, mitochondrial alteration and ER dilation. While we did observe an increase in the number of ER cisternae (both non- and dilated) near vs far from the lesion site (Far 13.65 ± 1.890 ER per MG/MDM vs Near 29.67 ± 3.531 ER per MG/MDM, *p* = 0.0003), the increase was attributed specifically to the non-dilated ER (Far 13.41 ± 1.887 non-dilated ER per MG/MDM vs Near 28.79 ± 3.383 per MG/MDM, *p* = 0.0003) as there were no significant differences in the dilated ER between the two regions (Far 0.2432 ± 0.08133 dilated ER per MG/MDM vs Near 0.8810 ± 0.2752 dilated ER per MG/MDM, *p* = 0.1389). The percentage of dilated ER over all ER observed was also similar when comparing far from vs near the lesion site (Far 2.187 ± 0.7448% of dilated ER vs 2.645 ± 0.7405% of dilated ER, *p* = 0.3804). This result suggests that the proximity to the injury site does not affect the proportion of dilated ER.

**Fig. 3:**
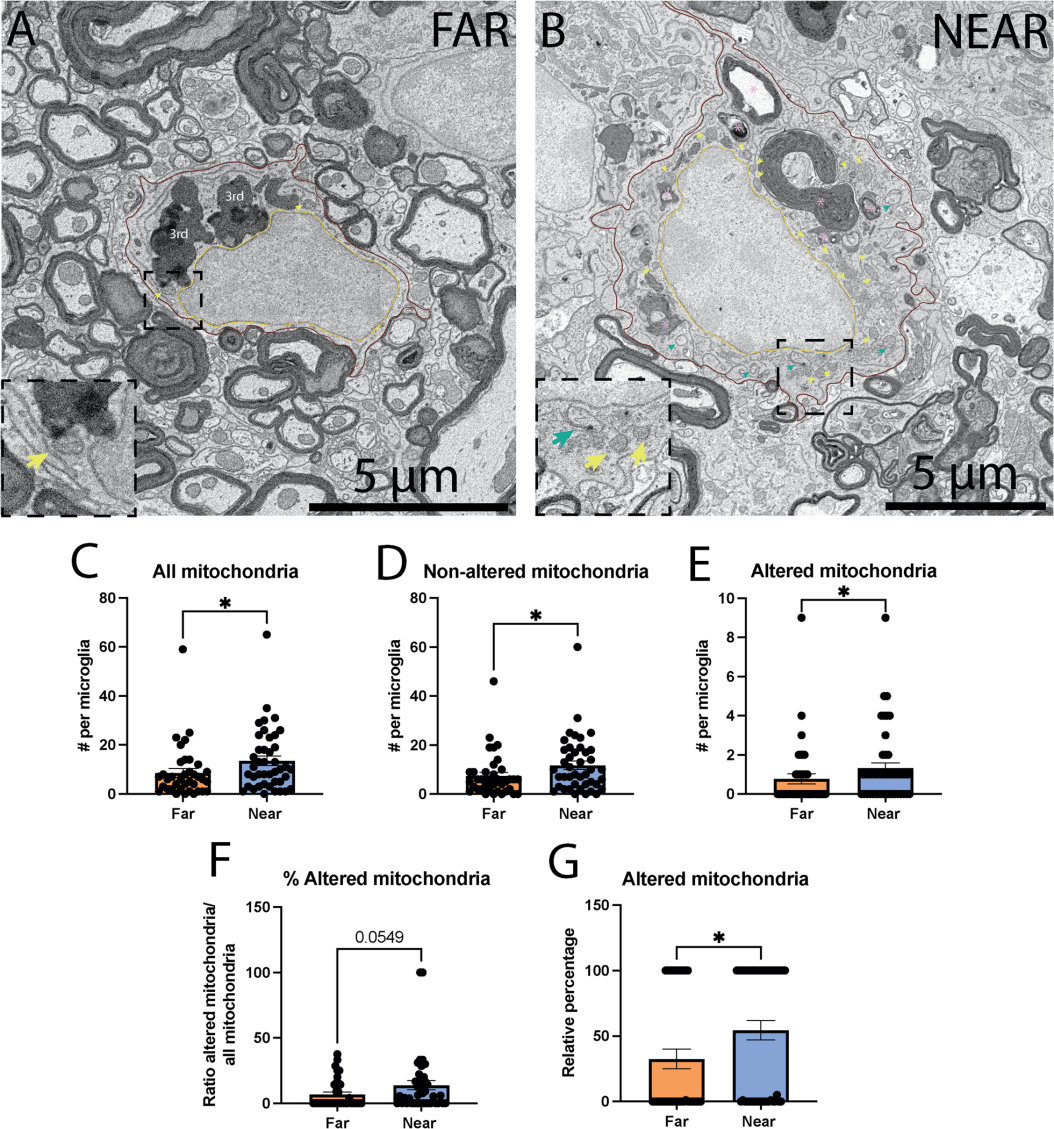
Mitochondrial characterization of MG/MDM far from and near the spinal cord injury (SCI) site in the dorsal column. Representative 5 nm per pixel scanning electron microscopy (SEM) images of MG/MDM far (**A**) and near (**B**) the SCI site in the dorsal column of lower thoracic spinal cord of 8-week-old female SCI mice. Quantitative graphs representing the number mitochondria (both altered and non-altered) per MG/MDM cell body (**C**), the number of non-altered mitochondria (**D**), the number of altered mitochondria (**E**), the ratio of altered mitochondria over all mitochondria (**F**) and the relative percentage of MG/MDM cell bodies that are positive for at least one altered mitochondrion within their cytoplasm (**G**). Data are expressed as individual dots and are shown as means ± SEM. *p < 0.05, using a non-parametric Mann–Whitney test. Statistical tests were performed on *n* = 10–14 MG/MDM per animal with *N* = 3 mice/group, for a total of 79 cell bodies analyzed. Red outline = cytoplasmic membrane, yellow outline = nuclear membrane, ma = non-degraded myelinated axon, dma = degraded myelinated axon, blue asterisk = fully digested phagosome, pink asterisk = myelin phagosome, 3^rd^ = tertiary lysosome, black dotted line = zoom in insert, yellow arrow = non-altered mitochondria, green arrow = altered mitochondria

We quantified the number of mitochondria within the cytoplasm of MG/MDM far from vs near the injury site and found a significant increase in mitochondria near the injury site (Far 8.622 ± 1.777 mitochondria per MG/MDM vs Near 13.50 ± 1.949 mitochondria per MG/MDM, *p* = 0.0223). We then assessed the health status of mitochondria (i.e., degradation of inner or outer membrane, swollen or degraded cristae) within MG/MDM far from vs near the injury site. Both non-altered mitochondria and altered mitochondria were significantly more numerous in the MG/MDM near compared to the ones far from the injury site (Far 7.459 ± 1.458 non-altered mitochondria per MG/MDM vs Near 11.76 ± 1.734 non-altered mitochondria per MG/MDM, *p* = 0.0311; Far 0.7750 ± 0.2594 altered mitochondria vs Near 1.326 ± 0.2711 altered mitochondria, *p* = 0.0302) (Fig. 3C, D). While we found an increase for both non- and altered mitochondria, this significant difference could be due to the increased MG/MDM cytoplasmic area measured (Fig. 1K). To determine if this increase in altered mitochondria was due to a larger MG/MDM area, we investigated the ratio of altered mitochondria over all mitochondria in the cytoplasm of MG/MDM far from vs near the injury site. The ratio of altered mitochondria showed a tendency toward an increase in MG/MDM near vs far from the injury site, highlighting that more altered mitochondria are present in MG/MDM nearby the injury site regardless of their increase in cytoplasmic area (Far 6.768 ± 1.805% altered mitochondria vs Near 13.76 ± 3.508% altered mitochondria,* p* = 0.0549) (Fig. 3F). In addition, more MG/MDM near the injury site contained at least one altered mitochondrion compared to those located far from the injury (Far 32.53 ± 7.497% of MG/MDM vs Near 54.50 ± 7.401% MG/MDM, *p* = 0.0232) (Fig. 3G; Table 2). As mitochondrial functions are tightly linked to their structural integrity, these changes in their ultrastructure point toward functional impairments [66], suggesting that MG/MDM with a high presence of altered mitochondria near the lesion site could have an impaired energy production. Overall, we showed that MG/MDM near the injury site possess more mitochondria, with a higher ratio increase in altered mitochondria, and more cells positive for these altered elements.

**Table 2:** Relative ultrastructural analysis of MG/MDM far from and near the SCI injury site in the lower thoracic dorsal column of 8-week-old SCI female mice

	Far SCI Mean ± SEM	Near SCI Mean ± SEM
Altered mitochondria (%)*	32.53 ± 7.497	54.50 ± 7.401
Dilated endoplasmic reticulum (%)	21.62 ± 6.861	33.33 ± 7.362
Association with degraded myelin (%)	40.54 ± 8.183	59.52 ± 7.666

*n* number, *a.u.* arbitrary unit

The p values of statistically significant tests are highlighted with an asterisk symbol. Data reported is shown as the percentage of cells positive for at least one of the elements analyzed and expressed as means ± SEM. *p < 0.05 using a Kruskal–Wallis test with a Dunn’s multiple comparisons post hoc test. Statistical tests were performed on *n* = 10–14 MG/MDM per animal with *N* = 3 mice/localization, for a total of 79 cell bodies analyzed

Finally, this change in mitochondrial ultrastructure was not accompanied by the appearance of dark MG/MDM, while the presence of glycogen granules was observed in very few MG/MDM near the injury (Table 1; Additional file 2: Fig. S2).

## Discussion

In this study, we investigated for the first time the ultrastructural features of MG/MDM located far from vs near a SCI injury, specifically examining their intracellular contents and parenchymal interactions, in 8-week-old C57BL/6 female mice with a T11 spinal cord contusion injury. While MG and MDM were previously shown to be key players in the sub-acute to sub-chronic period following SCI [57, 68, 69], there is limited information on their ultrastructural alterations in relation to their proximity to the lesion site, which can help provide insights into their functional involvement (*e.g.*, phagocytic characteristics, changes in energy production, interactions with the parenchyma) based on their proximity to the spinal lesion (Table 1).

Our findings revealed that MG/MDM near the lesion sites present altered parenchymal contacts with myelinated axons and synaptic elements. In particular, MG/MDM interacted less with synaptic elements near compared to far from the lesion site, while no difference in their direct contacts with non-degenerated myelinated axons was observed. Previous work identified a reduced density of cholinergic axon terminals at 6 weeks post injury in 12-week-old female mice with a T7–T9 SCI [70, 71], indicating that the reduced interaction with synaptic elements could be due to the loss of cholinergic terminals. It will be interesting in future studies to determine the neurotransmitter nature of the MG/MDM-contacted axon terminals.

Evans et al*.* previously observed frequent interactions between Thy1+/YFP axons and CX3CR1^+/GFP^ MDM in 8–20-week-old male and female THY1-^YFP^/CX3CR1^+/GFP^ mice with a dorsal column crush injury at 2, 5, and 8 days post-injury [57]. Destructive contact, defined by drastic reshaping of the myelinated axons, was specifically attributed to infiltrating MDM [57]. Infiltrating MDM were also shown to be the main cell type in contact with degenerated axons at 7 days post-injury in 8–14-week-old female lys-EGFP-knock in mice with a moderate T11 contusion injury [16]. We observed a tendency for an increase in the MG/MDM interactions with degenerated myelinated axons near compared to far from the lesion site. It is unknown if these contacts resulted in “destructive contact” as observed by Evans et al. [57]. In this respect, a limitation of our study is the combination of MG and MDM in our quantitative ultrastructural analysis, making it impossible to compare the distinctive characteristics of each cell type to isolate their specific roles. Future studies using fluorescent reporter mouse models would be warranted to examine these distinctive characteristics.

In addition, we further explored the impact of the proximity to the lesion core and observed a significant increase in the phagocytosis of myelinated elements in MG/MDM near compared to far from the lesion site. This is in line with previous work that identified myelin-containing phagosomes using electron microscopy in a specific microglial state (CD11c+) following peripheral nerve injury in male and female *Itgax-Venus* mice [59]. In addition, we found a significant increase in fully and partially digested phagosomes within MG/MDM located near compared to far from the lesion site. These results are also aligned with previous data demonstrating an increase in mRNA levels of *cd68*, a phagolysosomal activity marker [42], at 7 day post-injury in female adult Wistar rats following a T9 compression injury [72]. However, it should be noted that this elevated abundance of MG/MDM phagosomes could be due to an increase in their cytoplasmic area, as observed in MG/MDM located near compared to far from the lesion site in our 8-week-old SCI female mice. An enlarged or increased area of MG cytoplasm was previously reported in primary microglial cells from 8- to 12-week-old male C57BL/6 mice following a chronic constriction injury in the sciatic nerve vs sham surgery [73], and in male Sprague–Dawley with a T13 SCI [74].

In addition, in this study, we investigated the possible accumulation of glycogen granules, which were previously observed in MG near amyloid beta plaques and dystrophic neurites in 20-month-old APP–PS1 male mice [27], as well as the presence of dark MG, a microglial state associated with various markers of cellular stress [27, 29]. Dark MG where previously observed in high numbers in mouse models of maternal immune activation [poly I:C injection [44], high-fat diet [28]], chronic stress [29], Alzheimer’s disease pathology [27, 29] and aging [29], among other conditions. In our SCI mouse model, similar to what was observed in mice injected with lipopolysaccharide [43], we did not find dark MG. The lack of dark MG observed in this model could be due to the timing of the observations, 7 days after SCI. Previously, dark MG were not observed after an acute challenge with lipopolysaccharide, contrary to more chronic models of disease (e.g., Alzheimer’s disease, Huntington’s disease, amyotrophic lateral sclerosis pathology), suggesting that sustained challenges could be needed to increase their number [30, 31, 43]. In addition, as these cells were similarly not observed in mice after traumatic brain injury [75], their involvement in injury vs disease remains to be examined further. We also observed few instances of MG/MDM positive for the presence of glycogen granules, which were abundant in MG strictly nearby amyloid beta plaques and dystrophic neurites in aged APP–PS1 male mice [27], indicating that SCI at this time point does not strongly induce nor reduce in MG/MDM near or far from the injury site the presence of glycogen granules, contrary to what has been previously observed in MG near amyloid beta plaques and dystrophic neurites in a mouse model of Alzheimer’s disease pathology [27].

We also investigated the presence of cellular stress, notably dilated ER and structurally altered mitochondria, in MG/MDM located far from vs near the lesion site. We found an increased abundance of non-altered and altered mitochondria in the MG/MDM cytoplasm near the lesion site compared to far. This is in line with the literature which highlights the intimate relationship between mitochondrial damage and increased ROS levels [63, 67]. Compromised bioenergetic mitochondrial functions alongside increased oxidative stress was previously observed in adult female Sprague–Dawley with a T10 contusion [67]. In adult male Sprague–Dawley rats at 3 day post-injury following a C5 hemi-contusion, an increase in markers of oxidative stress was also observed [62]. Four and 14-month-old C57BL/6 female mice who underwent a contusion SCI compared to their male counterparts expressed higher levels of genes associated with ROS, such as *Nox2* [76], suggesting possible sex differences in the molecular mechanism taking place post-SCI. Further investigation into the ultrastructural alterations caused by differing ROS levels in SCI between males and females should be performed in the future.

## Conclusion

In summary, MG/MDM show different ultrastructural characteristics in relationship to their distance to the spinal cord injury site. We found that MG/MDM near the injury site have more mitochondria, and an increased number and proportion of mitochondria with morphological alterations. MG/MDM near the injury site displayed increased phagocytic activity, revealed by the increased number of phagosomes, including phagosomes with myelin and partially digested content. Finally, the interaction with synaptic elements of these cells was different far from and near the injury site. Our data suggests that MG/MDM near the injury have increase phagocytic activity and different metabolic requirements that could be key to understanding the biological processes post-injury.

### Supplementary Information

**Additional file 1: Figure S1.** Identification of lesion site. Representative 25 nm per pixel scanning electron microscopy (SEM) chip mapping image of the dorsal column of lower thoracic spinal cord of 8-week-old female spinal cord injury (SCI) mice (A). Lesion area showing ultrastructural signs of dystrophy, such as dystrophic axons with authophagosomal vesicles (B), apoptotic cells (C) and abundance of myelin alterations (D). The parenchyma area far from the lesions shows myelinated axons without signs of dystrophy (E) and cell bodies without signs of cellular stress (F, G). White dotted line = lesion area, black dotted line = zoom in inset.

**Additional file 2: Figure S2.** Microglia containing glycogen granules. Representative 5 nm per pixel scanning electron microscopy (SEM) image of MG/MDM positive for glycogen granules near the spinal cord injury (SCI) site in the dorsal column of lower thoracic spinal cord of 8-week-old female SCI mice. Red outline = cytoplasmic membrane, yellow outline = nuclear membrane, white arrow heads = glycogen granules, black dotted line = zoom in inset.

## Data Availability

All data presented in this study are available from the corresponding author upon reasonable request.

## Abbreviations

CNS: Central nervous system
CSF1R: Colony-stimulating factor 1 receptor
ER: Endoplasmic reticulum
IBA1: Ionized calcium-binding adaptor molecule 1
MG: Microglia
MDM: Monocyte-derived macrophages
PB: Phosphate buffer
PBS: Phosphate-buffered saline
PFA: Paraformaldehyde
ROS: Reactive oxygen species
SCI: Spinal cord injury
SEM: Scanning electron microscopy
