# Supplementary figures and images for: Microglia/macrophages are ultrastructurally altered by their proximity to spinal cord injury in adult female mice
Source: J Neuroinflammation. 2023 Nov 21;20:273. doi: 10.1186/s12974-023-02953-0 (PMC10664529; doi:10.1186/s12974-023-02953-0)

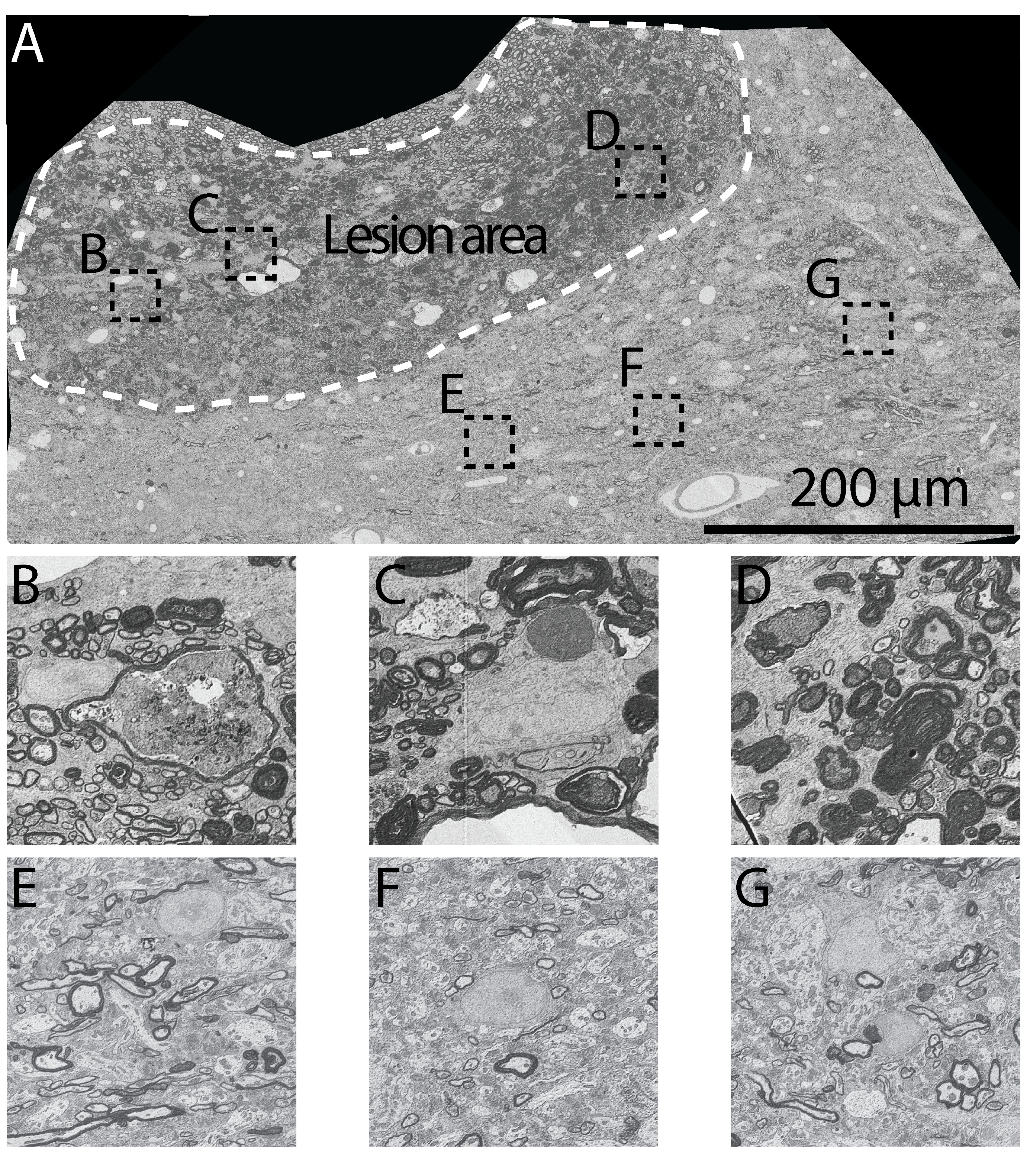

Supplement: Supplementary file 1 — Additional file 1: Figure S1. Identification of lesion site. Representative 25 nm per pixel scanning electron microscopy (SEM) chip mapping image of the dorsal column of lower thoracic spinal cord of 8-week-old female spinal cord injury (SCI) mice (A). Lesion area showing ultrastructural signs of dystrophy, such as dystrophic axons with authophagosomal vesicles (B), apoptotic cells (C) and abundance of myelin alterations (D). The parenchyma area far from the lesions shows myelinated axons without signs of dystrophy (E) and cell bodies without signs of cellular stress (F, G). White dotted line = lesion area, black dotted line = zoom in inset. [file 12974_2023_2953_MOESM1_ESM.tif]

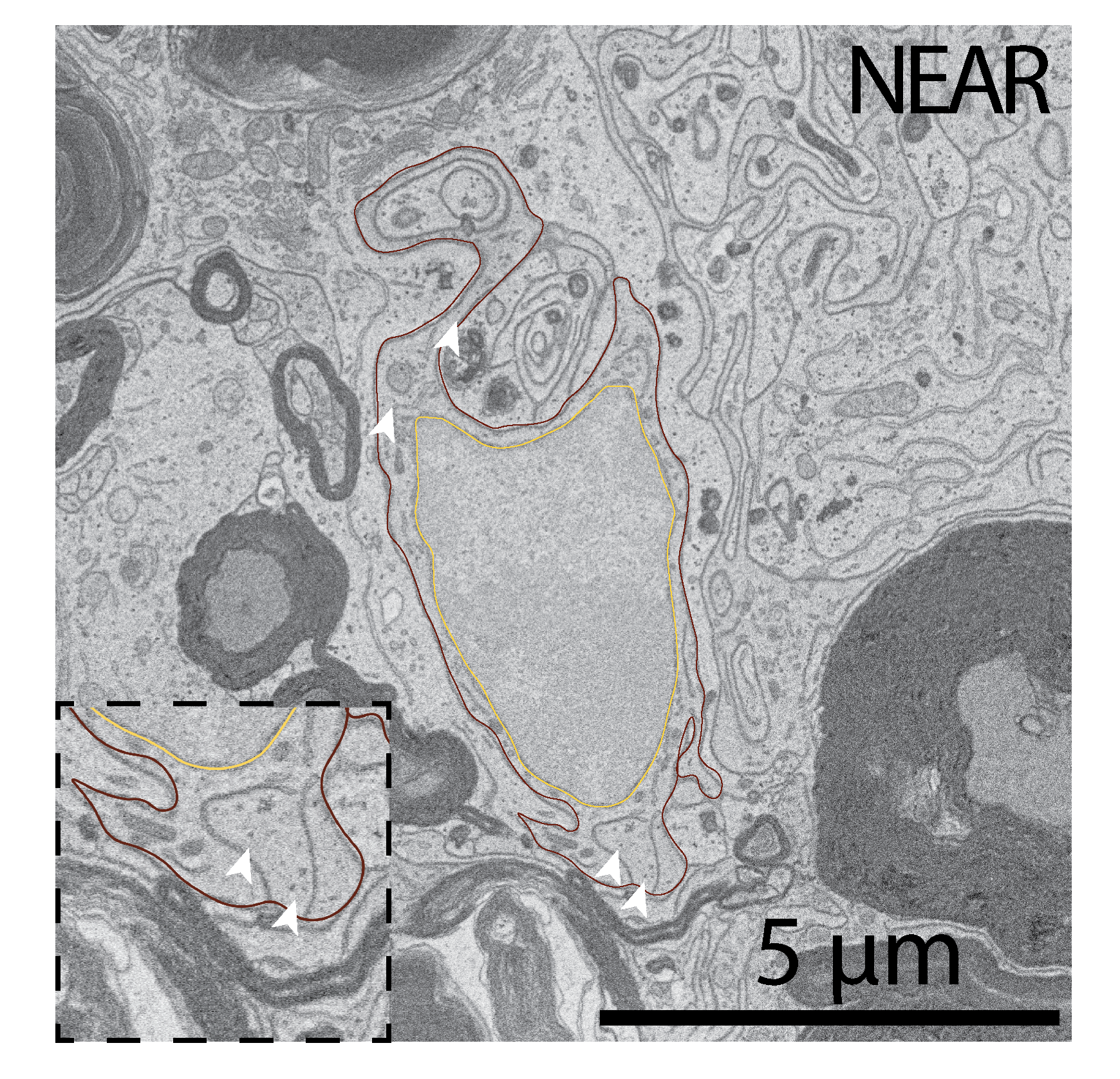

Supplement: Supplementary file 2 — Additional file 2: Figure S2. Microglia containing glycogen granules. Representative 5 nm per pixel scanning electron microscopy (SEM) image of MG/MDM positive for glycogen granules near the spinal cord injury (SCI) site in the dorsal column of lower thoracic spinal cord of 8-week-old female SCI mice. Red outline = cytoplasmic membrane, yellow outline = nuclear membrane, white arrow heads = glycogen granules, black dotted line = zoom in inset. [file 12974_2023_2953_MOESM2_ESM.tif]
